# Effects of lipid-probe interactions in biochemical fluorometric methods that assess HDL redox activity

**DOI:** 10.1186/1476-511X-11-87

**Published:** 2012-07-06

**Authors:** Theodoros Kelesidis, Srinivasa T Reddy, Diana Huynh, David Meriwether, Alan M Fogelman, Mohamad Navab, Otto O Yang

**Affiliations:** 1 Department of Medicine, David Geffen School of Medicine, University of California, Los Angeles, CA, 90095, USA; 2 Department of Microbiology, Immunology, and Molecular Genetics, David Geffen School of Medicine, University of California, Los Angeles, CA, 90095, USA; 3 Department of Obstetrics and Gynecology, David Geffen School of Medicine, University of California Los Angeles, Los Angeles, CA, 90095, USA; 4 Department of Molecular and Medical Pharmacology, David Geffen School of Medicine, University of California, Los Angeles, CA, 90095, USA; 5 Department of Medicine, 37-121 Center for Health Sciences, 10833 LeConte Avenue, Los Angeles, CA, 90095, USA

## Abstract

**Background:**

Fluorescence-based cell-free assays offer an attractive alternative to current cell-based assays for measuring the redox activity of High-Density Lipoprotein (HDL). We have recently developed a biochemical assay that assesses the effect of HDL on the oxidation rate of dihydrorhodamine 123 (DHR), reflected by increasing fluorescence over time. However, an immediate reduction in the fluorescence signal is observed after addition of HDL to DHR, due to fluorescence quenching from lipid-probe interactions. Understanding this process is important for interpretation of the results of all fluorescence-based cell-free assays that measure oxidative properties of lipids.

**Methods:**

We determined the effect of quenchers (proteins or lipids) on the fluorescence signal of two fluorescence-based cell-free assays: the rhodamine 123 (RHD)-based assay, and a previously described assay based on dichlorodihydrofluorescein (DCF) in patients with systemic inflammation or atherosclerosis versus healthy subjects.

**Results:**

We found lipid-probe interactions between the non-fluorescent substrate and the lipid, which affect the observed rate of change of fluorescence after addition of lipids to DHR and DCFH. These interactions depended on: sample collection and storage, types and concentrations of lipid and fluorescent probe, method of HDL isolation, diluents and matrices, and pH. The RHD-based assay yielded reproducible measurements despite fluorescence quenching, while the DCF-based assay displayed more experimental variability. Furthermore, the lipid-probe interactions varied according to the setting of systemic inflammation when using apolipoprotein (apo) B-depleted plasma. However, under fixed conditions the rhodamine assay could reliably detect similar mean relative differences in the redox activity of HDL samples between different groups of patients using either purified HDL or apo-B depleted plasma.

**Conclusions:**

Lipid-probe interactions should be considered when interpreting the results of fluorescence assays for measuring lipid oxidative state. Ideally, samples should be freshly obtained and purified HDL should be utilized rather than Apo B-depleted serum. Assay variability can be reduced by strict standardization of conditions (particularly sample collection, storage, lipid isolation method). Data comparisons between different studies similarly require strict standardization of conditions between studies and this caveat must be considered when using these assays to study the role of HDL function in the development of atherosclerosis *in vivo*.

## Introduction

There is a continuing search for new biomarkers of increased risk for atherosclerotic disease. High-density lipoproteins (HDL) function rather than absolute level may be a more accurate indicator for risk of developing atherosclerosis [1]. Measuring HDL cholesterol levels provides information about the size of the HDL pool, but does not predict HDL composition or function. Robust assays to evaluate the function of HDL are needed to supplement the measurement of HDL cholesterol levels in the clinic [2]. Currently, HDL functional properties are most often determined by cell-based assays [3-7]. However, the limitations of these cell-based assays render them impractical to scale up for large-scale clinical trials.

We previously developed a cell-free assay that measures HDL function by testing the effect of HDL on the production of reactive oxygen species (ROS) after oxidation and conversion of dichlorodihydrofluorescein diacetate (DCF-DA) to fluorescent DCF (2',7'-dichlorofluorescein) [3]. Although this assay yielded results that correlated to the cell-based assay, it has not found widespread usage due to technical difficulties in getting consistent results across other researchers. Thus, we recently developed an alternative cell-free biochemical assay to quantify the redox activity of HDL [8].

The new assay measures the products of redox cycling as the rate of oxidation of the fluorogenic probe dihydrorhodamine-123 (DHR) to fluorescent rhodamine 123 (RHD) [8,9]. Thus, the redox activity of HDL is assessed in terms of capacity to engage in redox cycling (1;8). This assay provides a readout that is highly correlated to a validated cell-based assay when using purified HDL [6,7,10], yet is reproducible, and amenable to high-throughput implementation.

A key factor in interpreting both cell-free assays of HDL is potential lipid-probe interactions affecting the fluorescent readout. We have observed that such interactions can affect the fluorescent signal independently of the redox effects of HDL, and thus could be confounders [8]. Thus, the aims of this study are to investigate lipid-probe interactions by testing lipids with different oxidative properties in the two different fluorescence-based cell free assays and to determine various factors that affect these lipid-probe interactions. We determined these effects using blood samples from subjects with atherosclerosis such as coronary artery disease (CAD) and from patients with systemic inflammation such as Human Immunodeficiency virus (HIV-1)-infected individuals. Understanding these biochemical interactions in fluorescent cell-free assays is important for using these assays as tools to measure functional properties of HDL.

## Materials and methods

### Subjects

Ten blood samples were randomly selected from pretreatment samples remaining from a previously described study in which all patients had coronary artery disease or equivalent defined by the National Cholesterol Education Program Adult Treatment Panel III criteria [11] as previously described [8]. 50 Human Immunodeficiency virus (HIV-1)-infected individuals on combination antiretroviral therapy with suppressed viremia (below 50 copies of RNA/ml) (48 males and 2 females; median age 44, range 18–53 years) were recruited at the University of California, Los Angeles (UCLA) as previously described [8]. Sixty normal volunteers (52 males and 8 females; median age 42, range 18–57 years) were recruited under a protocol approved by the Human Research Subject Protection Committee of the University of California, Los Angeles. Fifty of them were matched by age and sex to the 50 HIV-1 infected individuals.

### Reagents

Dihydrorhodamine 123 (DHR) and Dihydrodichlorofluorescein diacetate (DCFH-DA) were obtained from Molecular Probes (Eugene, OR). DHR was prepared as a stock of 50 mM in dimethyl sulfoxide (DMSO) and DCFH was prepared as previously described [8,12]. Iron-free HEPES (N-2-hydroxyethylpiperazine-N'-2-ethanesulfonic acid)-buffered saline (HBS, HEPES 20 mM, NaCl 150 mM, pH 7.4) was prepared as previously described [8,9]. The DHR stock was diluted 1:1000 in HEPES saline solution to prepare a working solution of 50 μM. Purified Apolipoprotein A-I from human plasma was obtained from Sigma-Aldrich (St. Louis, MO). Oxidized l-α-1-palmitoyl-2-arachidonoyl-sn-glycero-3-phosphorylcholine (oxPAPC) was prepared as previously described [3].

### HDL and LDL purification

HDL and Low-Density Lipoprotein (LDL) were isolated from cryopreserved human plasma by ultracentrifugation, Polyethylene glycol (PEG) precipitation and fast performance liquid chromatography (FPLC), aliquoted and stored as previously described [13-17]. HDL and LDL cholesterol were quantified using a standard colorimetric assay (Thermo DMA Co., San Jose, CA, USA) as previously described [3].

### DCF-based cell-free assay of HDL function

The DCF-based cell-free assay was performed as previously described (3;12). Kinetic determination of fluorescence was performed by measuring the rate (slope) of oxidation of DCFH over 50 minutes after addition of a specific amount of lipid (HDL, LDL) as previously described [8]. Endpoint determination of fluorescence at 50 minutes after addition of a specific amount of lipid (HDL, LDL and oxPAPC) was performed as previously described (3;8;12).

### RHD-based cell-free assay of HDL function

Quadruplicates of HDL (2.5 μg of cholesterol unless otherwise specified) were added to 96-well plates (polypropylene, flat bottom, black, Fisher Scientific, USA). HBS was added to each well to a final volume of 150 μl, followed by addition of 25 μl of the 50 μM DHR working solution, for a total volume of 175 μl (final DHR concentration of 7 μM). Immediately following DHR addition, the plate was protected from light and placed in the fluorescence plate reader (at 37 °C). The fluorescence of each well was assessed at two minute intervals over an hour with a Synergy 2 Multi-Mode Microplate Reader (Biotek, Vermont, USA), using a 485/538 nm excitation/emission filter pair with the photomultiplier sensitivity set at medium. Determination of oxidation rate of DHR was performed by measuring the slope of fluorescence increase over 50 minutes after addition of a specific amount of lipid (HDL, LDL) as previously described [8]. Endpoint determination of fluorescence at 0 and 50 minutes after addition of a specific amount of lipid (HDL, LDL and oxPAPC) was performed as previously described (3;8;12). HDL oxidative function was calculated as the mean of quadruplicates for the wells containing the HDL sample.

### Statistical analysis

Statistical analysis was performed using Excel (Microsoft Corporation, Seattle, WA) and STATA statistical software (version 12). Continuous variables were explored for normal distribution and normally distributed continuous variables were expressed as mean ± standard deviation and non-normally distributed continuous variables were expressed as median and interquartile range (IQR). The comparison of quantitative variables among 2 groups was done using the Student-*t* test for normally distributed variables and the Mann–Whitney *U* test for non-normally distributed variables. Correlations between different variables were calculated using the Pearson correlation coefficient or the non-parametric equivalent. Significance was set at p < 0.05 (2 tailed).

## Results

### Lipid-probe interactions affect the observed rate of fluorescence after addition of lipids to DHR

**Figure 1:**
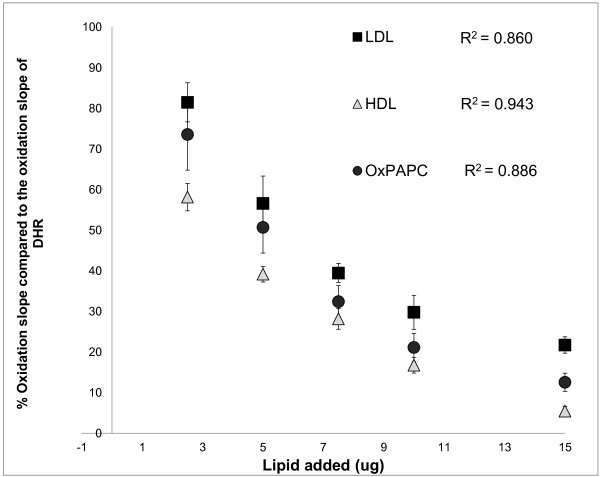
**Dose-dependent decrease in DHR oxidation rate (as measured by changes in fluorescence per minute over 50 minutes) by incubation with increasing concentration of HDL (triangles) and LDL (squares) that were isolated by FPLC from a healthy donor, oxPAPC (circles) that was prepared as described in Materials and Methods and stable amount of DHR (50 uM).** The slope of oxidation of each sample was normalized to the slope of oxidation of DHR and the % relative oxidation slope and quenching is shown on the y axis. The correlation between the relative % slope and the added amount of lipids is shown. The values represent means ± SD of quadruplicate samples from 3 independent experiments.

In our previously reported assay that measures the redox activity of HDL, we unexpectedly observed a significant reduction in fluorescence signal after initial addition of HDL [8]. To examine the specificity of this decrease, we compared the effect of addition of different lipids with known oxidative properties on the oxidation rate of DHR (Figure 1). LDL and L-α-1-palmitoyl-2-arachidonoyl-sn-glycero-3-phosphorylcholine (oxPAPC), both lipids known not to be anti-oxidant, lead to significant decrease in the rate of observed fluorescence after addition to DHR (Figure 1). Because these lipids are pro-oxidant, this suggested that lipid-probe interactions cause interference with the fluorescence readout.

### DHR and DCFH differ in their lipid-probe interactions

**Figure 2:**
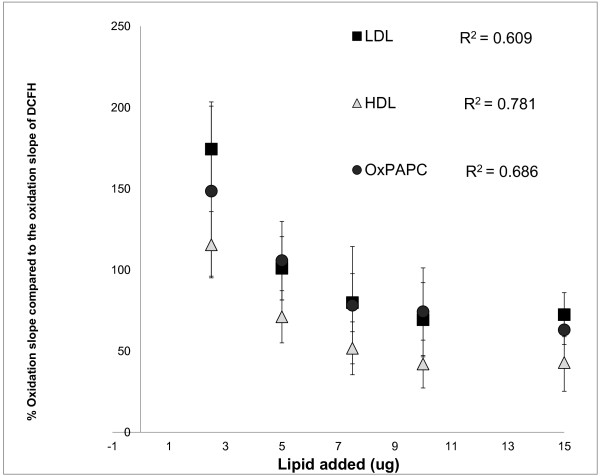
**Dose-dependent decrease in DCF oxidation rate (as measured by changes in fluorescence per minute over 50 minutes) by incubation with increasing concentration of HDL (triangles) and LDL (squares) that were isolated by FPLC from a healthy donor, oxPAPC (circles) that was prepared as described in Materials and Methods and stable amount of DCFH (200 ug/ml).** The slope of oxidation of each sample was normalized to the slope of oxidation of DCF and the % relative oxidation slope and quenching is shown on the y axis. The correlation between the relative % slope and the added amount of lipids is shown. The values represent means ± SD of quadruplicate samples from 3 independent experiments.

The same lipids were tested for their effects in the previously described Dihydrodichlorofluorescein diacetate (DCF) assay of HDL (3;12). HDL, LDL, and oxPAPC all lead to reduction in the DCF fluorescence and the fluorescence rate of DCFH-DA (Figure 2), but only at higher lipid concentrations (> 7.5 μg). However at lower lipid concentrations (< 5 ug) no reduction in the observed fluorescence was observed, in contrast to DHR (Figure 1). In addition the % fluorescence quenching (defined as reduction in the fluorescence signal of the fluorochrome immediately after addition of the lipid) that was observed with DCF at higher lipid concentrations (>7.5 ug) was less prominent compared to the % fluorescence quenching that was observed with DHR (Figure 1). Thus, the lipid-probe interactions in fluorescence-based cell free assays that determine oxidative properties of lipids depend on both the type of the fluorochrome used in the assay and the concentration of the lipid, and are more prominent with DHR compared to DCFH.

### Lipid-probe interactions depend on the concentration of the lipid

To determine whether the lipid-DHR interactions were dependent on the concentration of the lipid, increasing concentrations of cholesterol (HDL, LDL) and oxPAPC were incubated with 50 μM of DHR. The correlation observed between the quantity of the lipid added and DHR fluorescence and the oxidation rate of DHR (DOR) (Figure 1) was inverse and highly significant. Similarly, the lipid-DCF interactions were also dependent on the concentration of the lipid, and increasing concentrations of cholesterol (HDL, LDL) and oxPAPC lead to reduction in the DCF fluorescence and oxidation rate of DCFH (Figure 2).

### Lipid-probe interactions depend on the concentration of the fluorescent probe

**Figure 3:**
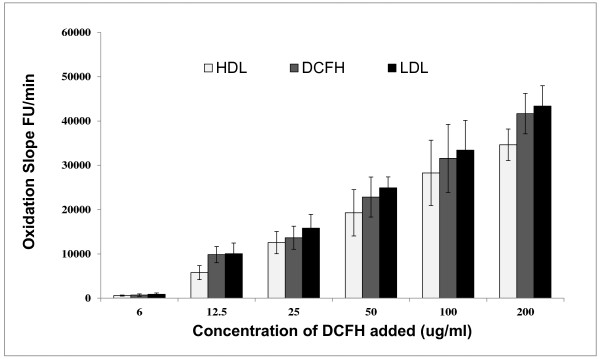
**Dose-dependent increase in DCFH oxidation rate (as measured by changes in fluorescence per minute) after incubation of specific amount (2.5 ug) of HDL (light gray columns), or LDL (solid black columns) that were isolated by ultracentrifugation from a healthy donor with increasing concentration of DHR (1, 10, 20, 30, 40, 50 uM).** Fluorescence intensity was determined after 1 h of incubation at 37° C. The values represent means ± SD of quadruplicate samples.

We previously showed that increasing concentrations of DHR (1–50 μM) resulted in increasing oxidation rate of both LDL and HDL, and that the oxidation rate of LDL was higher than that of HDL [8]. To further investigate the concentration dependent interaction between fluorescent probes and lipids, increasing doses of probes were added to specific amount of HDL or LDL (2.5 μg). To increase the sensitivity of the assay to detect smaller differences in oxidative properties of lipoproteins, we used 50 μM of DHR for all our experiments, which is a concentration that has been previously used to quantify redox activity [9]. Similarly, increasing concentrations of DCFH resulted in increasing oxidation rate of both LDL and HDL (Figure 3). Again no significant quenching was observed with DCF and lower amounts of lipids (2.5 ug) (Figure 2).

### Lipid-probe interactions occur between the non-fluorescent substrate and the lipid rather than the oxidized fluorescent product and the lipid

**Figure 4:**
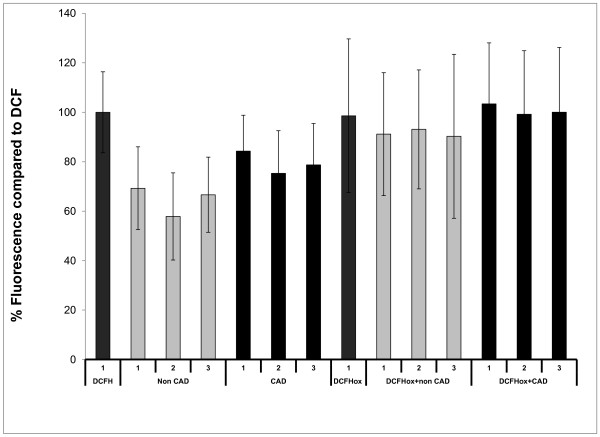
**Oxidation of DCFH in the presence of six different samples of HDL [3 from patients with coronary artery disease (CAD) and 3 from healthy volunteers (Non CAD)] was assessed as described in Methods, using 2.5 μg (cholesterol) of added HDL.** The slope of oxidation of each sample was normalized to the slope of oxidation of DCFH and the % relative oxidation slope and quenching is shown on the y axis. The data (means of quadruplicates) from three independent experiments are plotted. The experiments were repeated after DCFH (grey columns) was almost fully oxidized (air oxidation at room temperature for 5 hours). There was a ~40-fold increase in the slope of oxidation of DCFH after 5 hours of air oxidation. The oxidized DCF (DCF ox) was diluted 40-fold to give comparable fluorescence to the baseline DCFH and experiments after addition of HDL were repeated. Addition of HDL did not cause significant fluorescence quenching and there was a significant increase in the variability of oxidation of DCFH compared to baseline DCFH. These results indicate that most of DCFH had converted into DCF and did not react with HDL confirming the fact that fluorescence quenching is the result of DCFH-HDL interaction.

We then determined the nature of the lipid-probe interactions and whether the lipid interacts with the non-fluorescent substrate, the oxidized fluorescent product or both. When most of DHR was converted (oxidized) into rhodamine (RHD) the lipid-probe interactions were significantly less prominent, suggesting that fluorescence quenching is the result of DHR-HDL interaction rather than RHD-HDL interaction [8]. Similarly, when most of DCFH was converted (oxidized) into DCF, the lipid-probe interactions again were significantly less prominent, suggesting that fluorescence quenching is the result of DCFH-HDL interaction rather than DCF-HDL interaction (Figure 4). Thus, the lipid-probe interactions in fluorescence-based cell free assays that determine oxidative properties of lipids occur mostly between the non-fluorescent substrate and the lipid.

### Different methods of HDL isolation affect lipid-probe interactions

**Figure 5:**
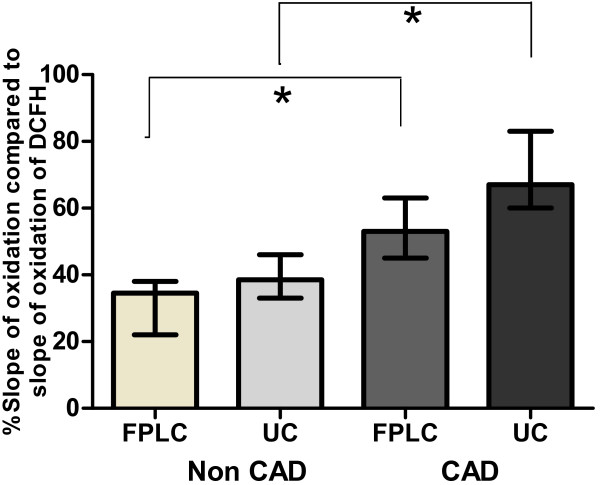
**Influence of method of HDL isolation on HDL-DCFH interactions. DCFH was exposed to 2.5 μg (cholesterol) of FPLC-purified (FPLC) or ultracentrifugation-purified HDL (UC) that was isolated from 10 patients with coronary artery disease (CAD) and 10 healthy volunteers (Non CAD) as described in Materials and Methods.** Each sample was run in quadruplicate. The median slopes of oxidation are expressed as % oxidation slopes compared to the median slope of oxidation of DCFH. The median oxidation rate of DCFH was significantly higher when HDL isolated by ultracentrifugation was added compared to when HDL isolated by FPLC was added (p < 0.05, n = 20). * p < 0.05.

Different methods of HDL purification may affect lipid-probe interactions and the readout of the RHD-based assay. The observed oxidation rate of DHR (DOR) was significantly higher when the HDL had been isolated by ultracentrifugation compared to HDL isolated by other methods such as fast performance liquid chromatography (FPLC) [8]. Similarly, the oxidation rate of DCFH was significantly higher when HDL isolated by ultracentrifugation was added compared to when the same amount of HDL isolated by FPLC was added (Figure 5).

### The concentration dependent effect of addition of HDL on oxidation of the fluorogenic probe is not secondary to lipid-free apolipoprotein AI (apoAI)

**Figure 6:**
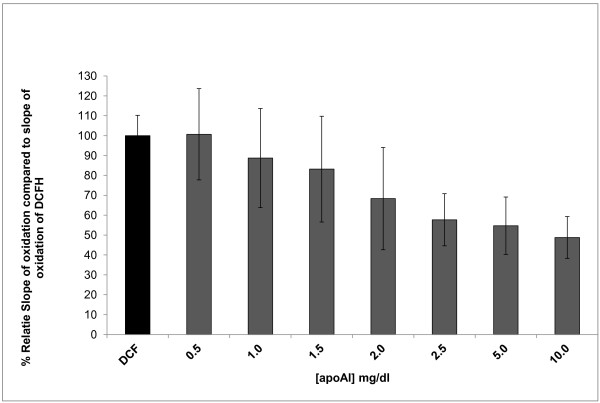
**The effect of purified, commercially available, lipid-free apolipoprotein AI (ApoAI) from a healthy donor on DCF oxidation rate was assessed by incubation of 200 ug/ml of DCFH with increasing concentration of ApoAI (0.5 -10.0 mg/dl).** The slope of oxidation of each sample was normalized to the slope of oxidation of DCF and the % relative oxidation slope and quenching is shown on the y axis as described in Figure 5A. The values represent means ± SD of quadruplicate samples. There was no concentration dependent effect of apoAI (at concentration > 2 mg/dl) on DCFH oxidation rate.

It has previously been shown that lipid-free apoAI has no concentration dependent effect on oxidation of DHR at concentrations that correspond to the physiologic range of concentration of apoAI in normal subjects (90–130 mg/dl) [3,8,18], Similarly, the addition of increasing concentrations of lipid-free apoAI (2.5-10 mg/dl) did not have a concentration dependent effect on oxidation of DCFH (Figure 6) confirming our previous results [3]. The protein (lipid free apoAI) -probe interactions were more prominent with DHR compared to DCFH [8] (Figure 6).

### Different diluents affect lipid-probe interactions

Different methods of HDL purification and storage utilize varying compositions of diluent [8]. Moreover, different types of diluents utilized for HDL suspension and storage in preparation for HDL functional assays can affect the readout of the RHD-based assay [8]. Specifically, use of sodium azide and/or standard phosphate-buffered saline (PBS) significantly increased fluorescence quenching and reduced oxidation of DHR compared to the buffered saline diluent (HBS) [8]. The effects of different diluents on lipid-probe interactions were confirmed in the DCF-based assay; addition of phosphate (PBS) and/or sodium azide significantly enhanced fluorescence quenching and increased experimental variability compared to use of HBS (data not shown). Of note, in the previous DCF-based assay the diluent that was used was PBS with sodium azide and this may also partially explain the increased experimental variability observed with that method (3;12). These results suggest that use of saline without sodium azide and PBS reduces lipid-probe interactions and improves the sensitivity of the assay to detect differences in oxidative properties of lipids between different samples.

### Changes in pH can affect lipid-probe interactions

It has previously been demonstrated that oxidation of DHR was also pH-dependent and that the oxidation rate of DHR was highest at pH 3, least at pH 9, and similar for pH 5 and 7 [8]. However in the DCF-based assay the oxidation rate of DCFH was highest at pH 9, least at pH 3, and similar for pH 5 and 7 (data not shown). These results suggested that the lipid-probe interactions are less prone to pH effects near the physiologic range and careful titration of the pH of the HBS diluent may reduce experimental variability.

### Matrix effects can affect lipid-probe interactions

**Figure 7:**
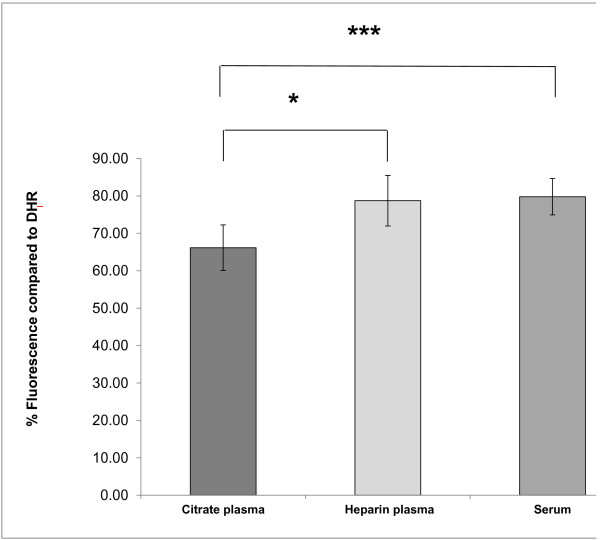
**HDL was isolated from plasma (with heparin or citrate as anticoagulants) and serum of 12 different healthy volunteers, using ultracentrifugation.** Oxidation of DHR in the presence of these 12 different HDL samples was assessed as described in Methods, using 2.5 μg (cholesterol) of added HDL. The slope of oxidation of each sample was normalized to the slope of oxidation of DHR and the % relative oxidation slope and quenching is shown on the y axis. The data (means of quadruplicates) from three independent experiments are plotted. We found that the HDL samples isolated from plasma with heparin and from sera had significantly (p < 0.05) higher DOR values (78.7% ±6.8 and 79.8 ± 4.9 respectively) compared to the same HDL samples isolated from plasma citrate (66.2% ±6.1).

**Figure 8:**
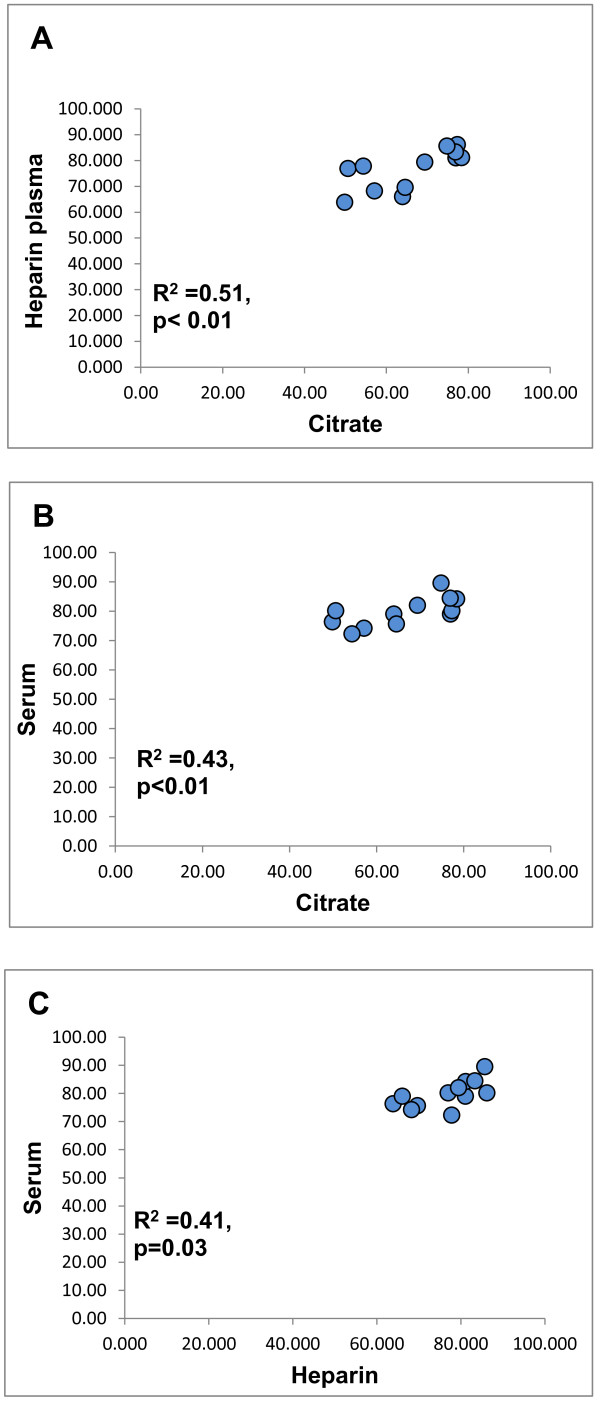
**HDL was isolated from plasma (with heparin or citrate as anticoagulants) and serum of 12 different healthy volunteers, using ultracentrifugation.** Oxidation of DHR in the presence of these 12 different HDL samples was assessed as described in Methods, using 2.5 μg (cholesterol) of added HDL. The slope of oxidation of each sample was normalized to the slope of oxidation of DHR and the % relative oxidation slope and quenching is shown on the y and x axes. The data (means of quadruplicates) from three independent experiments are plotted. We found that the DOR values from the HDL samples isolated from plasma with heparin correlated significantly (p < 0.05) with those from the HDL samples isolated from sera (R^2^ = 0.41, p = 0.03) or plasma citrate (R^2^ = 0.51, p < 0.01). The DOR values from the HDL samples isolated from plasma with citrate correlated significantly (p < 0.05) with those from the HDL samples isolated from sera (R^2^ = 0.43, p < 0.01).

We determined the effects of different matrices (plasma with citrate versus plasma with heparin versus serum) on the rate of oxidation of DHR (DOR). We found that the HDL samples isolated from plasma with heparin and sera had significantly (p < 0.05) higher DOR values compared to the same HDL samples isolated from plasma citrate (Figure 7) but the DOR values correlated significantly (R² = 0.51, p < 0.01; R² = 0.43, p < 0.01 respectively) (Figure 8).

### Differences in factors that affect lipid-probe interactions may partially explain the increased experimental variability observed with the DCF-based assay compared to the RHD-based assay

**Figure 9:**
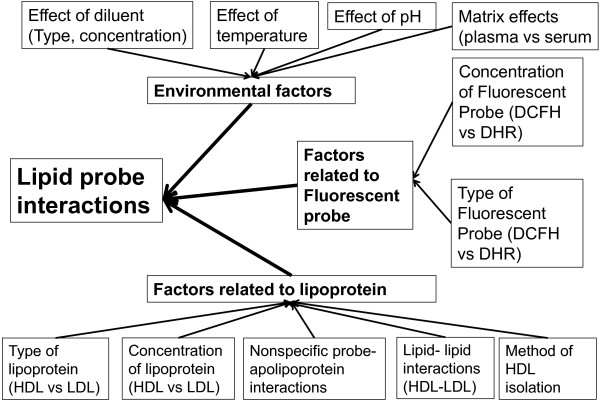
**Summary of factors that affect lipid-probe interactions.** The RHD-based assay has reduced experimental variability compared to the DCF-based assay. This can be partially explained by the following differences between the RHD-based assay and the DCF-based assay a) Use of a single lipid (HDL) in the RHD-based assay versus 2 lipids (HDL and LDL) in the DCF-based assay b) DHR is relatively stable, and oxidizes at a predictable rate when exposed to air compared to DCFH which is more unstable and prone to auto-oxidation c) Formation of DCFH from DCFH-DA can be mediated by esterases that are carried over variably during the lipid purification process, while DHR requires no activation and is not prone to this effect d) Use of HBS buffer rather than PBS/azide e) Lipid-DHR interactions are less affected by changes in pH compared to DCFH-lipid interactions.

We observed a significantly higher experimental variability (as assessed by larger error bars) in the DCF-based assay compared to the RHD-based assay (Figures 1,2,3,4,5,6,7,8) [8]. Figure 9 summarizes the factors that can affect lipid-probe interactions and that can partially explain this increased experimental variability of the DCF-based assay compared to the RHD-based assay.

### The clinical status of study subjects can affect the degree of lipid-probe interaction when apolipoprotein (apo) B-depleted plasma is assayed in the RHD-based assay

**Figure 10:**
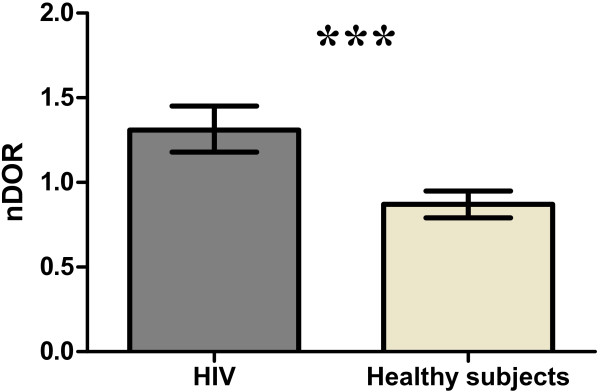
**HDL was isolated from plasma of 50 patients with HIV infection and 50 healthy subjects, using ultracentrifugation and PEG precipitation as described in Methods.** Oxidation of DHR in the presence of these 100 different HDL samples was assessed as described in Methods, using 2.5 μg (cholesterol) of added HDL. The slope of oxidation of each sample was normalized as ratio to the DOR of a control pool of HDL isolated from plasma of 50 healthy control subjects using ultracentrifugation and PEG precipitation as described in Methods. The normalized DOR (nDOR) is shown on the y axis. The data (means of quadruplicates) from three independent experiments are plotted. The median nDOR value from HIV-infected subjects was significantly (p < 0.001) higher (1.31; IQR: 1.18, 1.45) compared to the median nDOR value from healthy subjects (0.87; IQR: 0.79, 0.95) when HDL was purified using ultracentrifugation. This study had ≥ 85% study power to detect a 50% difference of nDOR (as a measure of HDL function) between patients with systemic inflammation (such as HIV infected subjects) and healthy subjects using non-parametric tests for independent samples at alpha 0.05 (two-sided).

**Figure 11:**
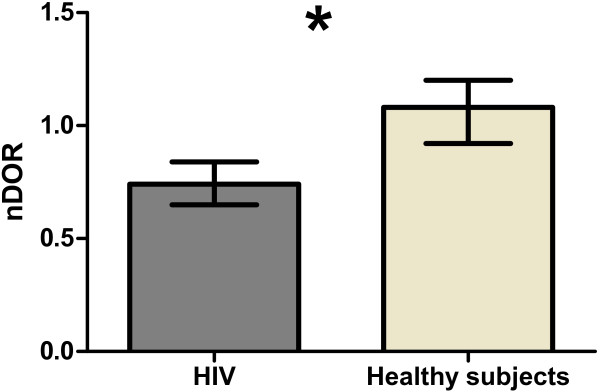
**Contrary to when HDL was purified using ultracentrifugation (Figure**10**), the normalized median oxidation rate of DHR (nDOR) was significantly (p < 0.05) lower in patients with HIV (0.74; IQR: 0.65, 0.84) compared to healthy subjects (1.08; IQR: 0.92, 1.20) when (apo) B-depleted serum was used.**

**Figure 12:**
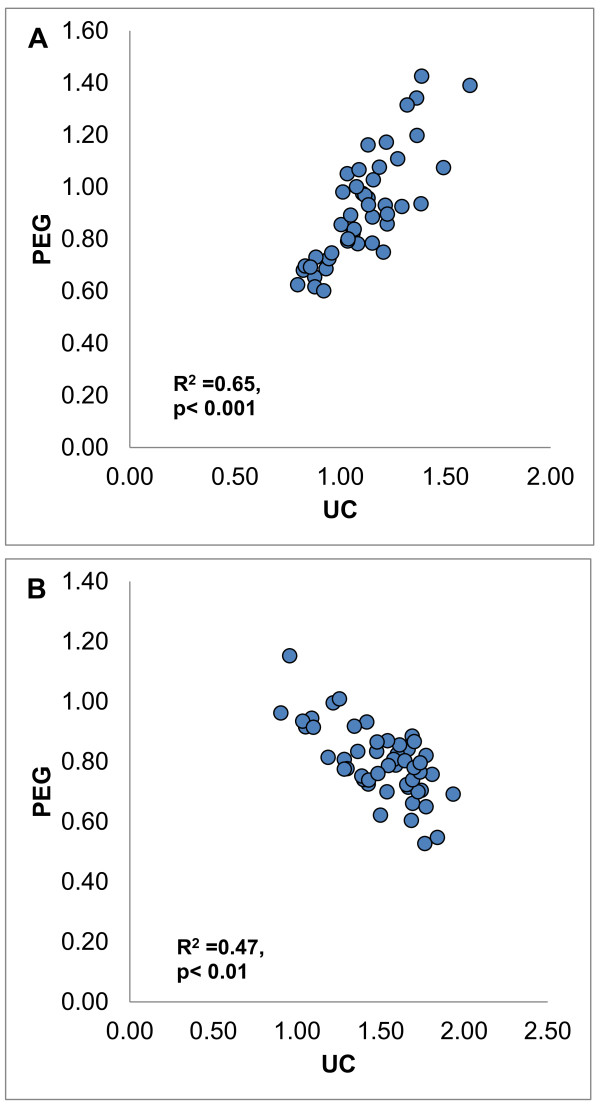
**HDL was isolated from plasma of 50 patients with HIV and 50 healthy subjects, using ultracentrifugation and PEG precipitation and DOR values were determined as described in Figure**10**.** We found that the nDOR values from the HDL samples isolated using ultracentrifugation (UC) correlated significantly (p < 0.05) with those from the (apo) B-depleted plasma using PEG precipitation in both healthy subjects (A) (R^2^ = 0.65, p < 0.001) and HIV infected (B) (R^2^ = 0.47, p < 0.01)

Although the RHD assay may allow high throughput study of HDL in the setting of large scale studies [8], the limiting factor is HDL isolation, prompting consideration of convenient approaches such as PEG precipitation [19]. We determined (apo) B-depleted plasma-DHR interactions for HIV-1-infected subjects (systemically inflamed) versus healthy subjects, using HDL that had been isolated by ultracentrifugation versus PEG precipitation (Figure 10). The oxidation rate of DHR (DOR) was significantly higher (p < 0.05) in the group with HIV-1 infection compared to healthy subjects when ultracentrifuged HDL was assayed (Figure 10), but lower when (apo) B-depleted cryopreserved plasma was used (Figure 11). Within groups, the relationships of DOR measurements using PEG-precipitated versus ultracentrifuge purified HDL were consistent, but these relationships differed between groups (Figure 12). This suggested that other plasma factors provoked by inflammation and or cryopreservation that remained in the apo-B depleted plasma but not in ultracentrifuged HDL interfere with the fluorescence readout [20].

## Discussion

Recent interest has focused on the functional consequences of HDL oxidation [1,21]. Oxidation conceivably could contribute to the formation of dysfunctional HDL, proposed to be involved in human cardiovascular disease [22-24]. Levels of reactive oxygen species (ROS) such as lipid hydroperoxides that are produced from oxidation of lipoproteins are significantly associated with functional properties of HDL [3]. To study these issues, fluorescence-based reporter assays have been applied to probe protein-protein interactions and monitor levels of ROS [25-29]. The generation of ROS can be followed as an increase in fluorescence that can be detected by different fluorochromes such as DCF [3] and rhodamine 123 (RHD) [9]. In the DCF-based cell-free assay the function of HDL is determined based on the capacity of HDL to prevent the formation or to inactivate oxidized phospholipids produced by LDL [3]. However, technical difficulties with this assay have limited its utility. Thus, we recently developed a novel cell free assay that is based on the oxidation rate of the fluorochrome dihydrorhodamine 123 (DHR) as a surrogate marker of the functional properties of HDL, which is more robust compared to the previous DCF-based assay [8].

Fluorescence, however, is an environmentally sensitive phenomenon [25,30]. Environmental factors may de-excite the fluorophore, and/or quench fluorescence [25,30]. Quenching of fluorescence is defined as loss of fluorescence signal due to short-range interactions between the fluorophore and the local molecular environment [25,30]. Fluorophores exhibit changes in fluorescence due to environmental factors such as pH, solvent, presence of quenchers (such as proteins or lipids) that can cause concentration dependent decreases in fluorescence readout [25,30]. The effect of apolipoproteins and lipids on fluorescent probes (lipid-probe interactions) has minimally been studied [31]. Understanding the lipid-probe interactions in the setting of cell-free biochemical assays that are based on fluorescence to determine functional properties of HDL (such as the DCF-based and RHD-based assays) is important for interpretation of the results of these functional assays.

Herein, we identify factors that are important for lipid-probe interactions and that may increase experimental variability in fluorescence-based assays that quantify oxidative properties of lipoproteins. Factors related to the lipoprotein (type and concentration of lipoprotein, presence of apolipoproteins, purification method of lipoprotein, HDL-LDL interactions), fluorescent probe (type and concentration of probe) and environmental factors (type and concentration of diluent, temperature, pH, matrix effects) all can affect the fluorescence signal and lipid-probe interactions (Figure 9).

Firstly, factors related to the lipoprotein such as the type and concentration of lipoprotein, lipid-lipid interactions presence of apolipoproteins, method of isolation of lipoprotein, play a major role in lipid-probe interactions. In the previous DCF-based assay we demonstrated lipid-probe interactions between the fluorochrome DCFH with various lipids such as HDL, LDL, oxidized l-α-1-palmitoyl-2-arachidonoyl-sn-glycero-3-phosphorylcholine (PAPC), 1-palmitoyl-2-oxovaleryl-sn-glycero-3-phosphorylcholine (POVPC), hydroperoxyoctadecadienoic acid (HPODE) [3]. Here we confirm the lipid-probe interactions between DCFH and LDL and oxPAPC and demonstrate that increasing concentrations of these lipids lead to changes in the fluorescence signal, and increasing concentrations of HDL lead to a decrease in the fluorescence signal (HDL-DCFH interactions), consistent with our previous results [3].

We subsequently studied the interactions of lipids with DHR. We found that addition of lipids, including pro-oxidant lipids contained in lipoproteins, lead to significant decrease in oxidation signal of DHR. It has been demonstrated that the interaction of DHR-HDL is responsible for the fluorescence quenching in the RHD-based assay [8]. Similarly to the HDL-DCFH interactions increasing concentrations of HDL lead to a decrease in the fluorescence signal of RHD. However, the RHD-based assay of HDL redox activity yields highly reproducible measurements despite fluorescence quenching [8]. In addition, the inverse correlation between HDL concentration and oxidation rate of DHR allows quantification of functional properties of HDL using very low concentrations of HDL (≤2.5 ug per sample). Thus, using this assay we were able to quantify relative differences in the redox activity of different HDL samples that correlate with their functional properties [8].

We also found that addition of two lipids can lead to increased experimental variability in determination of the fluorescence signal compared to the highly consistent results seen with HDL alone [8]. Co-incubation of stable amount of DHR (50 uM) with different concentrations of two lipids simultaneously (HDL with LDL, HDL with oxPAPC) so that the total amount of lipid was fixed at 5 μg increased the variability of the assay (as determined by the standard errors in quantification of the fluorescence signal) compared to when the same amount (5 μg) of each lipid was incubated with DHR independently [8]. Similar results were observed with the DCF-based assay (data not shown). Thus, based on these results, we avoided co-incubation of two lipids in the novel RHD-based assay that was based on the interactions of only one lipid (HDL) with a fluorescent probe [8].

The lipid-probe interactions are mostly caused by interaction of the non-fluorescent probe (e.g. DHR) with lipid. We demonstrate that when the non-fluorescent probe (e.g. DHR) has been mostly converted into fluorescent probe (e.g. RHD), the lipid probe interactions are minimal. Moreover, addition of detergent (SDS) in the DHR + HDL reaction causes an increase in fluorescence (data not shown), consistent with a reduction in quenching due to dissociation of lipid-dye complexes [8]. It has also been shown that the reduction in the fluorescence signal of RHD after addition of HDL occurs immediately, when minimal oxidation of DHR has occurred and minimal amount of RHD is present [8]. This indicates that the immediate reduction in fluorescence after addition of the lipid is secondary to lipid probe interactions (DHR-lipid interactions). Finally, using LC/MS/MS analysis it has been shown that differences in fluorescence after addition of same amount of different HDL samples correspond to real differences in the degree of oxidation of DHR [8].

Different methods of HDL purification such as ultracentrifugation, fast performance liquid chromatography (FPLC), use of dextran sulfate and precipitation with polyethylene glycol (PEG) may affect lipid-probe interactions [8]. The oxidation rates of DHR and DCFH are significantly higher for HDL isolated by ultracentrifugation versus other methods [8]. However, the lengthy process of ultracentrifugation may yield additional ROS, contributing to this observation [32]. Removal of serum albumin bound to HDL by ultracentrifugation may also alter the association of nonpolar and polar substances including ROS associated with lipoproteins [32],42]. Thus, although results using different methods of HDL isolation may correlate significantly [8], ideally a consistent method of HDL isolation for all the samples in one study should be used when measuring relative differences in the oxidative properties of HDL between these different samples.

We determined the effect of lipid-free apolipoprotein A-I, the major apolipoprotein of HDL, on the fluorescent probes. It has previously been shown that, in contrast to apoJ, coincubation of apoA-I with PAPC plus HPODE did not prevent the oxidation of PAPC by HPODE and did not prevent the increase in DCF fluorescence [3]. Consistent with these results we demonstrated that addition of lipid-free apoAI did not cause a concentration dependent effect on oxidation of both DHR and DCFH at concentrations that correspond to the physiologic range of concentration of apoAI in normal subjects (90–130 mg/dl) (1;18). Thus, the fluorescent probes used in biochemical assays of HDL function quantify relative differences in the redox activity of HDL that cannot be attributed to apolipoprotein A-I.

Moreover, factors related to the fluorescent probe such as the type and concentration of probe play a major role in lipid-probe interactions. Indeed, the lipid-probe interactions in the DCF-based and the RHD-based assays are distinct. Differences in the chemical structure and/or charge between DHR and DCFH can explain these differences since DHR is positively charged while DCFH is negatively charged [33-41]. In contrast to DCFH, DHR has less complicated matrix effects than DCFH, is less prone to auto-oxidation, is unaffected by esterases that may influence the fluorescence signal independently of oxidation, and does not form a nonfluorescent lactone when the oxidation product is exposed to a pure lipid environment [33-41].

Environmental factors (type and concentration of diluent, temperature, pH) can also affect the fluorescence signal and lipid-probe interactions. Changes in fluorescence that were observed with different buffers are likely due to an effect of the buffer on the HDL complex and hence the interaction between the buffer and the fluorescent indicator. In the previous DCF-based assay the buffer was phosphate buffered saline (PBS) containing sodium azide (pH 7.4), whereas the RHD assay utilized HEPES-saline buffer (pH 7.4) to quantify redox activity of DHR [9]. Azide and PBS reduced the oxidation rate of DHR and increased its variability, and increased the quenching effect of lipids. Thus, we conclude that PBS and azide should be avoided in cell-free fluorogenic methods.

We also examined the effect of pH on the interaction between lipids and oxidation of fluorescent probes. The effect of pH on the lipid-probe interactions was different in the DCF-based compared to the RHD-based assay but in both assays there was reduced variability in oxidation rate of the fluorescent probes at pH of 7.4 compared to extremes of pH, suggesting that this is the optimal pH for these assays.

We also determined the effects of different matrices (plasma versus serum) on the fluorescence readout and DOR. We found that the HDL samples isolated from heparinized plasma or serum had significantly higher DOR values compared to the same HDL samples isolated from plasma citrate, although the DOR values correlated significantly between different matrices. Such differences between serum and plasma measurements could be, at least in part, related to products from the clotting cascade found in serum that may affect oxidation reactions. Our observation that heparin may interfere with determination of ROS using fluorescent probes are consistent with previous studies [32], suggesting that plasma from citrate-treated blood or serum may be preferable for the RHD assay.

Polyethylene glycol (PEG) precipitation is a convenient, reproducible, and rapid method to extract HDL from patient serum [19], which would be ideal for high throughput fluorogenic HDL assays. However, while PEG precipitation removes apo B-containing particles [19], apo-B depleted plasma contains a significant amount of HDL-associated proteome that can be altered during inflammatory conditions [20] and cryopreservation and can cause protein-probe interactions in fluorescence-based biochemical assays that measure redox activity of HDL. The interactions of apo-B depleted serum and DCFH were not prominent in the previous DCF assay [3]. Consistent with our results that protein-DCFH interactions are less prominent compared to DHR-protein interactions, Patel et al. demonstrated that a significant reduction in the fluorescent signal of DCF is present only at higher concentrations of apo-B depleted serum [19]. However, contrary to when HDL was purified using ultracentrifugation, the DOR was significantly lower in patients with HIV infection compared to healthy subjects when (apo) B-depleted plasma from cryopreserved samples was used. Alterations of the proteome present in the apo-B depleted plasma (but not in purified HDL) that is associated with HDL and which is upregulated during inflammatory conditions [20] and cryopreservation may enhance protein-probe interactions and may explain these results.

In conclusion, fluorescence based cell-free assays offer an attractive alternative to current cell-based assays for measuring functional properties of HDL, and may be more precise because they measure an important biochemical process underlying the biological phenotype. We demonstrate that the reduction in the oxidation signal that is observed after addition of HDL to fluorochrome such as RHD and DCF is secondary to fluorescence quenching and lipid-probe interactions. Thus controlling for lipid-probe interactions that are observed in these assays is important for interpretation of the results of these assays and comparisons of results within and between studies.

## Abbreviations

apoAI, Apolipoprotein AI; DCF, Dichlorodihydrofluorescein; DCF-DA, Dichlorodihydrofluorescein diacetate; DHR 123, Dihydrorhodamine 123; DMSO, Dimethyl sulfoxide; FPLC, Fast performance liquid chromatography; HDL, High-Density Lipoprotein; LC/MS, Liquid Chromatography/Mass Spectrometry; LDL, Low-Density Lipoprotein; HEPES, N-2-hydroxyethylpiperazine-N'-2-ethanesulfonic acid; PBS, Phosphate-buffered saline; oxPAPC, Oxidized l-α-1-palmitoyl-2-arachidonoyl-sn-glycero-3-phosphorylcholine; PAPC, l-α-1-palmitoyl-2-arachidonoyl-sn-glycero-3-phosphorylcholine; POVPC, 1-palmitoyl-2-oxovaleryl-sn-glycero-3-phosphorylcholine; HPODE), Hydroperoxyoctadecadienoic acid; pHDL, Proinflammatory HDL; RHD, Rhodamine 123; ROS, Reactive oxygen species.

## Competing interests

AMF, STR and MN are principals in Bruin Pharma and AMF is an officer in Bruin Pharma. This manuscript is related to the Patent UCLA Case 2011-774-1.

## Authors’ contributions

TK, DH, DM carried out the experiments and drafted the manuscript. TK, OY conceived of the study, participated in the design of the study and drafted the manuscript. SR, AMF, MN helped to draft the manuscript. All authors read and approved the final manuscript.
